# Live *E. coli* bacteria label-free sensing using a microcavity in-line Mach-Zehnder interferometer

**DOI:** 10.1038/s41598-018-35647-2

**Published:** 2018-11-21

**Authors:** Monika Janik, Marcin Koba, Anna Celebańska, Wojtek J. Bock, Mateusz Śmietana

**Affiliations:** 1 Photonics Research Center, Université du Québec en Outaouais, 101 Rue St Jean Bosco, Gatineau, QC J8X 3x7 Canada; 2 The National Institute of Telecommunications, Szachowa 1, Warszawa, 04-894 Poland; 3 The Institute of Microelectronics and Optoelectronics, Warsaw University of Technology, Koszykowa 75, Warszawa, 00-662 Poland

## Abstract

The paper presents the first study to date on selective label-free biosensing with a microcavity in-line Mach-Zehnder interferometer induced in an optical fiber. The sensing structures were fabricated in a single-mode fiber by femtosecond laser micromachining. In contrast to other studies of this sensing scheme, where only the sensitivity to refractive index changes in the cavity was investigated, this research used chemical surface treatment of the sensor to ensure detection specificity. Immobilized MS2 bacteriophages were applied as recognition elements specifically targeting live *E. coli* C3000 bacteria. It is shown that the sensor allows for real-time monitoring of biological phenomena taking place on the surface of the microcavity. The developed biosensor exhibits ultrahigh refractive index sensitivity of 15,000 nm/RIU and is capable of detecting live *E. coli* bacteria concentrations as low as 100 colony forming units (CFU)/mL in liquid volume as low as picoliters.

## Introduction

Nowadays early and fast pathogenic detection and identification are crucial, e.g., for patients’ examinations in hospitals, especially those with sepsis^1^ or infections, where the strain of bacteria has to be identified as soon as possible to find the best suitable treatment. They are also needed for diagnosis of the state of water reservoirs, biomedical research, as well as for safety in the food industry and environmental monitoring. Bacterial infections cause several millions of diseases worldwide each year^2^. Thus, researchers focus on biosensors directly detecting whole bacteria cells, rather than isolated biological components, e.g., enzymes^3^, lipopolysaccharides^4^, or outer membrane proteins^5^. This approach has several advantages: whole cells are more tolerant of environmental changes such temperature, pH, than isolated structures; Isolation of whole microorganisms from natural sources is very easy; There is no need of extensive preparation of the sample before measurements.

Currently, bacteria detection still relies mostly on classical microbiology methods including isolation and bacteria growth in selective media^6^. These techniques are very effective, but they require high technical skills and are time-consuming due to the slow process of microorganism culturing. Therefore, there is an urgent need to develop alternative bacteria detection methods. Over recent years, biosensor development tackled this problem and has adopted numerous modern approaches including polymerase chain reaction (PCR), genomic sequencing, mass spectrometry, and microarrays. However, these methods involve sophisticated and expensive equipment, require high technical skills and complicated multi-step sample preparation and post-processing.

To overcome limitations of existing technology a new biosensor needs to be highly sensitive, specific, cost-effective, compact and easy to use. Based on these expectations, optical fiber sensors can be a compelling alternative to conventional analytical techniques. These sensors have many advantages including immunity to electromagnetic interference, real-time detection, the possibility of multiplexing, and high resistance to harsh environmental conditions. A number of compact optical fiber sensors have been reported, including devices based on long-period fiber gratings (LPFGs)^7^, fiber Bragg gratings (FBG)^8^, and photonic crystal fiber (PC)^9^. LPFGs were shown to be especially effective for bacteria detection^4,10,11^. It is notable that in most of the studies, killed bacteria were applied instead of vivid microorganisms. Although the LPFG is compact and limits of detection (LOD) have been reported as low as 10^2^ Colony Forming Units (CFU)/mL^12^, this type of sensor with 5–6 cm length usually requires at least 500 µL of examined sample.

Since in most biological and chemical analysis only limited volumes of samples are available, therefore, significant attention has been focused on structures based on in-fiber microcavities. One of the reported devices is microcavity in-line Mach-Zehnder interferometer (µIMZI)^13^. The structure is constituted by a small and deep hole, micro-machined in a single-mode optical fiber. Light propagating through the fiber core splits at the cavity’s sidewall into two parts – one propagates in the core, and the other penetrates the cavity. The beams interfere with the second wall of the cavity. In this sensing scheme, the investigated media can directly interact with the fiber core. The µIMZI device offers very high refractive index (RI) sensitivity reaching over 20,000 nm/RIU, making it one of the most RI-sensitive devices to date^14^. Due to very precise fabrication, the device is small, highly reproducible, and portable. Moreover, it is temperature-insensitive^15^, which is essential since temperature is one of the most disrupting factors in RI measurements. Along with an examination of ultra-small volume samples, the µIMZI can certainly be used for label-free sensing concept. This concept relies mainly on changes in optical properties at the sensor’s surface. In comparison to fluorescent-based detection, in the label-free protocol, there is no need to use special tags or labeling, what enables us to detect the biological molecules in their natural form. Hence label-free sensing is usually cheaper, faster and easier to perform.

Some highly sensitive sensors based on µIMZIs have been proposed for sensing strain^16^, pressure^17^ and RI^14^, but never for specific biosensing, including bacteria detection. Recently Li *et al*. reported bovine serum albumin (BSA) µIMZI-based biosensor^18^. Although, the sensor exhibited high RI sensitivity reaching 10,000 nm/RIU, and the low detection limit, the specific receptor has not been applied. Thus, the recorded spectrum dependent on the RI of BSA solution which changes tighter with its concentration.

To adapt a device for pathogen detection, the sensor’s surface must be specially modified. One of the crucial steps for the efficiency of a biosensor is the choice of bioreceptor. The bioreceptor can have an impact on the sensitivity and can make the sensor specific towards the chosen bacterial target. To date, a broad spectrum of bioreceptors has been used for bacterial detection, including antibodies^19^, sugars/lectins^20^, proteins^4,11^, aptamers^21^, and phages^7,22^. Each of these recognition elements has its advantages and disadvantages. In particular, bacteriophages can be highly specific towards bacteria, are cost-effective, have a long shelf-life, and exhibit high thermal stability. Until now, T4 bacteriophages have been the leading choice for application on an LPFG for *E. coli* detection^7,22^. However, the MS2 bacteriophage is also known to infect the male *E. coli* bacterium. This virus has already been immobilized on an LPFG-based biosensor^12^. Due to the lysogenic cycle of MS2^23^ - which does not result in immediate destruction of the host cell in comparison to the T4 phage - at controlled conditions stable measurements were possible. Furthermore, its simple structure compared to the T4 phage assures that the phage is well-orientated on the fiber surface, what was very challenging in the case of the T4 application.

In this work, we present for the first time a bacteria-specific µIMZI-based label-free biosensor. The measurements are performed using different concentrations of live *E. coli* C3000 as well as bacteria dry weight. The MS2 phages are immobilized on the functionalized surface as specific recognition elements. This paper shows that besides tracking the changes in the RI of the liquid filling the cavity, the µIMZI-based sensor can also simultaneously investigate the phenomena taking place at its surface.

## Materials and Methods

### µIMZI fabrication and analysis

Structures in the form of cylindrical cavities with a diameter (d) of 50 µm were fabricated in standard Corning SMF28e fibers following the method described in^14^. In order to identify the response of the structures to a thin high-RI film, such as biological film on the cavity surface, a set of µIMZIs was coated by atomic layer deposition (ALD) with a high-RI aluminum oxide (Al_2_O_3_) film according to the procedure reported in^24^. The Al_2_O_3_ thickness reached *t* = 154 nm. The use of a nano-coating with a high-RI material made it possible to simulate the effects of biofilm formation. The deposition was followed by slow chemical etching of the film using 10 mM sodium hydroxide (NaOH) (Sigma-Aldrich) at 20 °C for a specific time, and the optical response was verified after extensive cavity washing with deionized water. During the entire process, the µIMZI transmission was monitored with an NKT Photonics SuperK COMPACT supercontinuum white light source and a Yokogawa AQ6370C optical spectrum analyzer in the spectral range of 1100–1700 nm. A set of water/glycerin solutions with RI varied in the range of n_D_ = 1.3330–1.3900 RIU was used to perform the reference RI sensitivity measurements. The RI of the solutions was measured using a digital refractometer VEE GEE PDX-95.

### Preparation of biological samples

All the bacteria strains, as well as the MS2 bacteriophages used in described experiments, were obtained from the INRS-Institute Armand-Frappier Research Center, where were prepared following the procedure described in^23^.

### Biofunctionalization of the µIMZI

**Figure 1:**
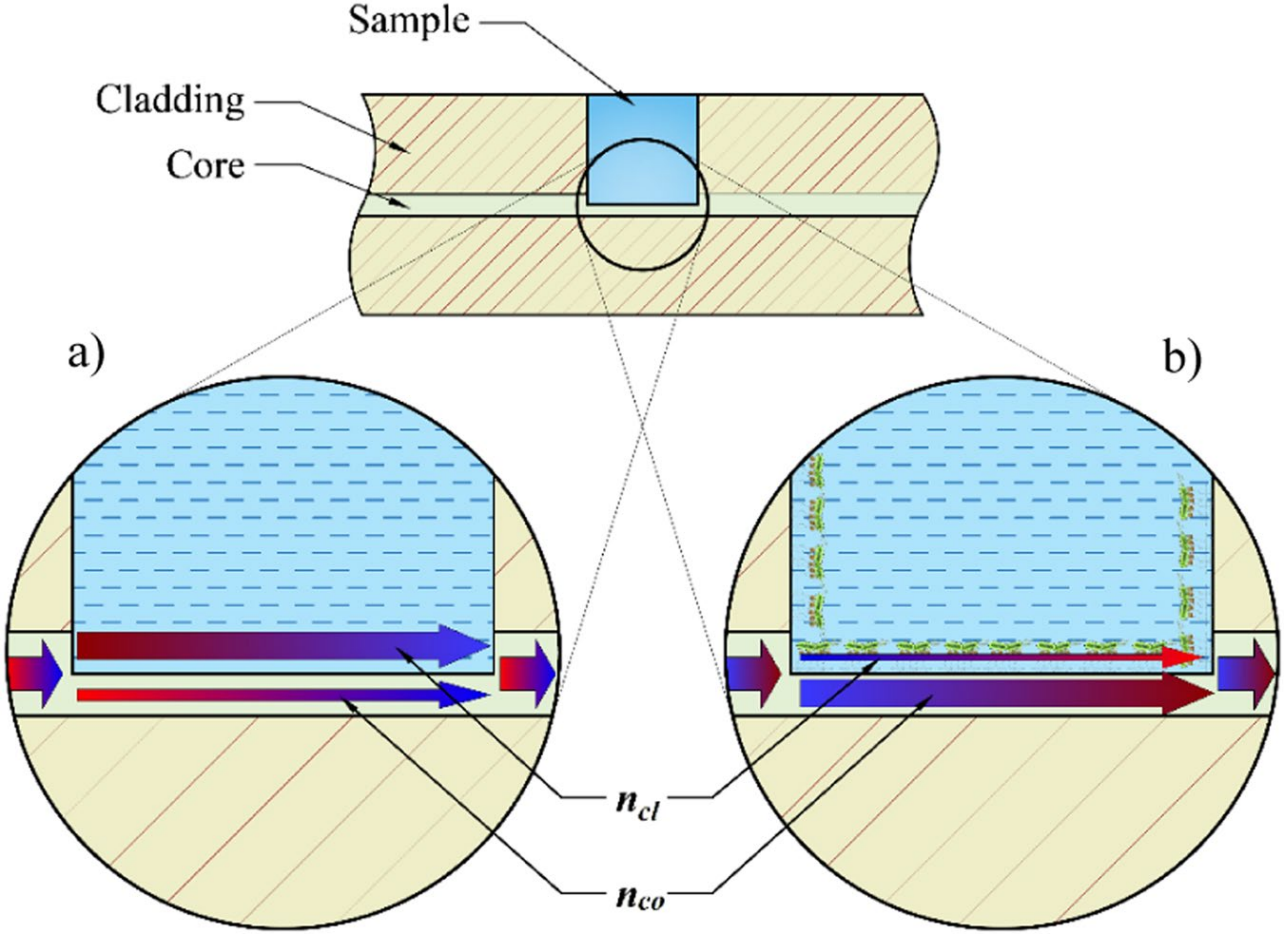
Schematically shown μIMZI structure and two interfering modes, namely *n*_*co*_, and *n*_*cl*_ in the remaining part of the fiber core and micromachined circular cavity, respectively, influenced by A. the liquid inside the cavity and B. specifically bound bio-layer. The drawing is not to scale.

In the first step of the functionalization of the surface, the cavity was cleaned with a mixture of hydrochloric acid and methanol (1:1, v/v) (Sigma-Aldrich) for 30 minutes and then in sulfuric acid (Sigma-Aldrich) (30 minutes) to remove contaminations and to enhance the density of superficial hydroxyl groups (Si-OH)^25^. This step was completed by extensive rinsing with water and vacuum drying. In the next step, the silanization process was carried out from the (3-Aminopropyl) triethoxysilane (APTES) (Sigma-Aldrich) gas phase according to the modified procedure presented in^26^. The effectiveness of the process is controlled by exposure time or the amount of the precursor. The silanization was performed in a small vacuum desiccator filled with argon. Two trays of a 30 µl of APTES and 10 µl of triethylamine (Sigma-Aldrich) (a sol-gel process catalyst) were placed in the desiccator chamber and left for over 30 minutes. The vials were then removed, and the cavity was kept in an argon atmosphere for 48 hours for curing^27^. Next, the deposited amine groups were activated by the homo-bifunctional cross-linking agent glutaraldehyde (GLU) (Sigma-Aldrich) through immersion in 2.5% GLU solution in PBS for 30 minutes. The obtained surface was very sensitive to amine groups at the capsid of the bacteriophages. For the conjugation, the sensor was incubated in a phages solution for 1 hour, followed by immersion in 2 mg/mL BSA solution in PBS for 30 minutes in order to block non-specific interaction. In the last step, the sensor was exposed to the bacteria for 30 minutes. The µIMZI sensor with MS2 phages immobilized on the surface was used to detect different concentrations of live *E. coli* C3000 strain in solution (Fig. 1). Bacteria dilutions in concentrations of 10^2^, 10^4^, 10^6^, 10^7^ and 10^8^ CFU/mL in PBS were prepared. For selectivity measurements, a negative sample containing *B. Thailandensis* was prepared in a concentration of 10^8^ CFU/mL. During experiments, the sensor was immersed in increasing concentration of C3000 strain for 30 minutes in each concentration. After the bacteria incubation, the sensor was extensively washed and measured in PBS to obtain reference results. The difference between wavelength corresponding to transmission minima after BSA binding and after each bacteria exposer was treated as the sensor response.

For determination of sensor’s selectivity, the µIMZI after immobilization of the MS2 phage and blocking with BSA was immersed in a solution of a non-specific strain of bacteria for 30 minutes, then washed and measured in PBS. Another selectivity verification was performed using *E. coli* C3000 with no MS2 phage immobilized but only blocked with BSA to exclude unspecific binding between bacteria and the functionalized surface.

## Results and Discussion

### RI sensitivity of the µIMZI sensor

**Figure 2:**
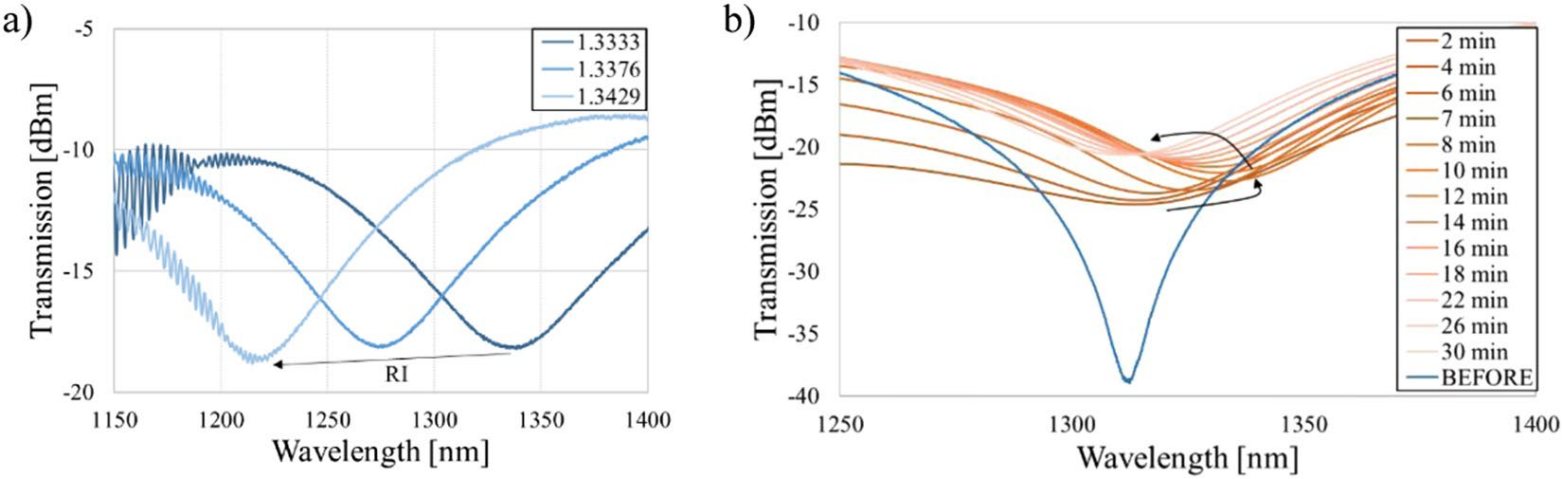
Transmission spectra of the µIMZI with the cavity diameter d = 50 µm, where (**a**) shows a response to RI changes and (**b**) shows the evolution of the Al_2_O_3_ film thickness (initially *t* = 154 nm) with its etching process recorded for a water-filled cavity. The spectrum before the deposition was given for reference. Arrows indicate the shift of the minimum induced by each procedure.

Before measuring µIMZI with bacteria, we studied the response of the sensor to different RI filling the cavity and thicknesses of the thin high-RI film deposited inside the microcavity. Following the analysis presented in^18,28^ and the Eq. (1) describing the spectral placement of the transmission minimum (*λ*_*m*_):

1 $${\lambda }_{m}=\frac{2\pi d\,{\rm{\Delta }}{n}_{eff}}{(2m+1)\pi -{\phi }_{0}},$$

where *Δn*_*eff*_ = *n*_*co*_ *−* *n*_*cl*_ is the difference between the effective RI of the two interfering modes, namely *n*_*co*,_ and *n*_*cl*_ in the remaining part of the fiber core and micromachined circular cavity, respectively, *d* is the diameter of the microcavity, and *φ*_0_ represents initial phase, it can be stated that the difference *Δn*_*eff*_ is the main component responsible for the spectral shift of the pattern (i.e., transmission minimum). Two different effects can induce the changes in *n*_*eff*_, namely changing the RI filling the cavity what corresponds mainly to *n*_*cl*_ or/and by the formation of an adlayer on the microcavity’s surface what in turn mainly influences *n*_*co*_. To investigate the response of the µIMZI to these two effects, reference measurements for different RI filling in the cavity and different thickness of the film on cavity surface were conducted (Fig. 2). In Fig. 2a is shown the effect of an increase in the RI in the microcavity. The transmission minima shift towards shorter wavelengths, and the RI sensitivity reaches 15,000 nm/RIU. In contrast, in Fig. 2b the response of the µIMZI to changes in thickness of a high-RI layer (Al_2_O_3_) deposited inside the cavity is shown. It has been determined using spectroscopic ellipsometry that the etching rate of Al_2_O_3_ reached ~0.65 nm/min^29^. The RI of Al_2_O_3_ is higher than that of either the fiber core or the cladding. Measurements were performed in water in consecutive time steps as shown in Fig. 2b. This figure illustrates that deposition of the thin layer caused a change in the depth of the resonance minimum from about −39 dB to −24 dB. Compared to the effect presented in Fig. 2a, the spectra do not change in a predictable, monotonous manner. During the first 10 minutes of the etching process, the transmission minimum shifts towards longer wavelengths. Later, as the thickness of the high-RI film decreases, a shift towards shorter wavelengths is observed. Depending on the µIMZI working conditions determined by the thin-film properties (mainly thickness and RI) and the depth of the microcavity, the effect of the changes in *n*_*co*_ manifests differently.

The results shown above indicate that both RI changes in the liquid and additional layer deposition have a significant influence on the µIMZI response. However, each of these parameters affects *Δn*_*eff*_ differently. When the RI of the liquid increases, the propagation conditions are affected primarily through the *n*_*cl*_, while depositing a high-RI film on the core surface influences mainly the *n*_*co*_^28^. This explains why we are observing the different behavior of the spectrum and allows us to interpret further results.

### Bacteria dry-weight sensing

One of the most significant differences between live bacteria and bacteria dry weight is that the lyophilized cells have no motility. Along with the incubation during measurements, cells limply sediment and aggregate on the sensor surface. This makes them easier to detect than the live, motile pathogen. Therefore, we perform a preliminary experiment with lyophilized *E. coli* C3000.

As shown in Fig. 2a, the spectral response of the µIMZI strongly depends on the RI filling the microcavity. That is why each step of the biofunctionalization was followed by measurements in PBS after extensive washing of the cavity. The purpose of washing was to remove biological residues which were only physically attached to the functionalized surface and could have a disturbing impact on the measurement. To achieve the relative shift of the transmission minima, only measurements performed in the same solution (here PBS) were compared.

**Figure 3:**
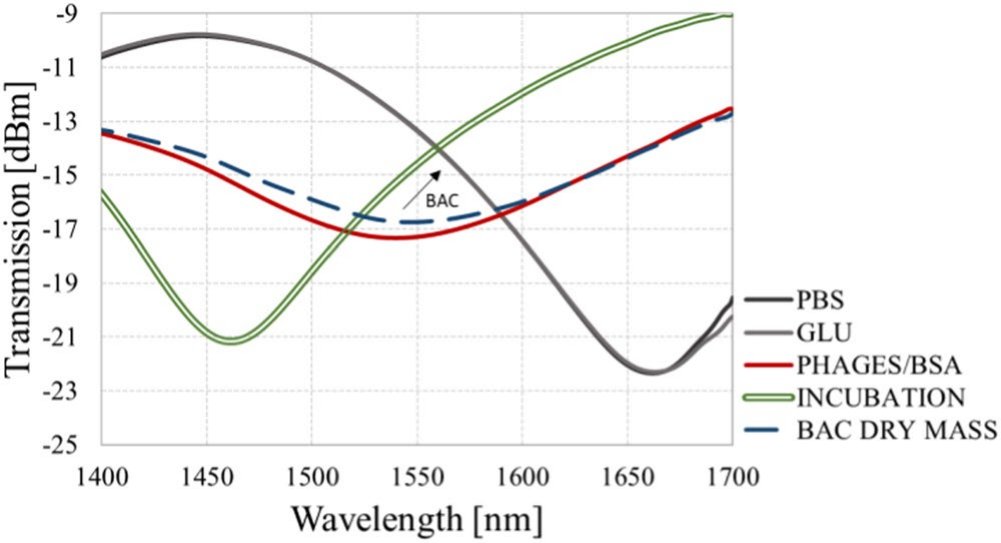
Transmission spectra of the µIMZI at each stage of the experiment with *E. coli* C3000 (1 mg/mL) dry weight. The arrow indicates the change induced by bacteria binding.

Figure 3 presents the transmission spectra obtained for each stage of the experiment, namely:

1. Functionalization of the surface, i.e., silanization with APTES (gas phase) (blue curve), where the process was controlled by exposure time and the amount of the precursor, followed by activation of the deposited amine groups by glutaraldehyde (GLU) (grey curve);
2. Immobilization of bioreceptor, i.e., MS2 phages and blocking the surface with BSA solution (red curve);
3. *E. coli* bacteria detection: incubation of the sensor in bacteria solution (green, double curve) and bacteria attached to the surface (blue, dashed curve).

Stage (1) for cavity filled with PBS can be considered as a baseline. What has been reported in^30^ thick layers of silane are very fragile and may be washed away either in the presence of a buffer or during various washing steps in an assay process. Therefore, the functionalization process was optimized to obtain a thin and stable silane/GLU layer, that could not change the working conditions of the µIMZI (no spectral shift of the pattern after the functionalization was noticed). On the other hand, the introduction of highly concentrated phages (10^9^ PFU/mL) (stage 2) with an average RI equal to 1.217 RIU^31^ changed working conditions of the µIMZI through the formation of low RI adlayer and thus significantly affected the *n*_*co*_. Presence of tens of nm thick layer had a significant influence on the spectrum, i.e., the minimum (λ_m_) strongly shifted towards lower wavelengths and decreased in depth from −23 dB before phages immobilization to −17 dB after the process. A similar effect can be observed in Fig. 2b, which shows how an additional layer caused a decrease in the intensity of the transmission. During the blocking step, the introduction of dense blocking solution (2 mg/mL) filled empty spaces and prevented from unspecific binding but did not introduce any further changes in λ_m_. This may be caused by the very small size of BSA molecules and an already uniform (dense) layer of phages. After the sensor surface functionalization, it has been exposed to bacteria dry weight (1 mg/mL) (stage 3). As can be seen in the Fig. 3, the minimum shifts 7 nm towards longer wavelengths (the shift is designated by a blue, dashed line). Given that the observed change induced by bacteria cells was obtained after extensive washing with PBS, the shift must be influenced by the growth of the specific adlayer on the microcavity surface, not by a change in the RI of the solution as observed during bacteria incubation (designated by the double green line).

### Detection of live *E. coli*

On the top of the lack of mobility, there are also other differences between killed and live microbes which can have an impact on measurement performance. After lyophilization, heat treatment or ultrasonication, the structural properties of bacteria cells are changed. Together with dehydration, changes may occur in the shape of the bacteria cell and in some of the surface structures, especially pili or fimbria, which are very important in the case of MS2-based detection. As a result, it is impossible to determine what has bonded to the surface – whole bacteria cells or some detached elements. Furthermore, during the sample preparation of killed microorganisms, bacteria cells can be destroyed, causing the release of internal structures such as proteins. This can also affect the optical responses. To imitate bacteria detection in their natural environment an experiment with live bacteria was conducted. Vivid bacteria are motile, in their proper shape, equipped with all the usual surface structures thus, makes measurements more challenging. A biolayer formed by live bacteria typically has different properties than layer previously created by bacteria dry-weight, including those optical. For example, it may contain air gaps due to the presence of fimbria/pili o the surface which holds the surface of the cells at a distance from the µIMZI bottom. Since immobilized MS2 are bound to the pili, this causes no problems with capturing bacteria and keeping them on the µIMZI surface. However, the layer can never be as dense and uniform as a layer formed by immobilized, killed bacteria or bacteriophages. What is more, the layer can scatter the light even more through the extremely rough surface of bacteria, what can lead to additional losses.

**Figure 4:**
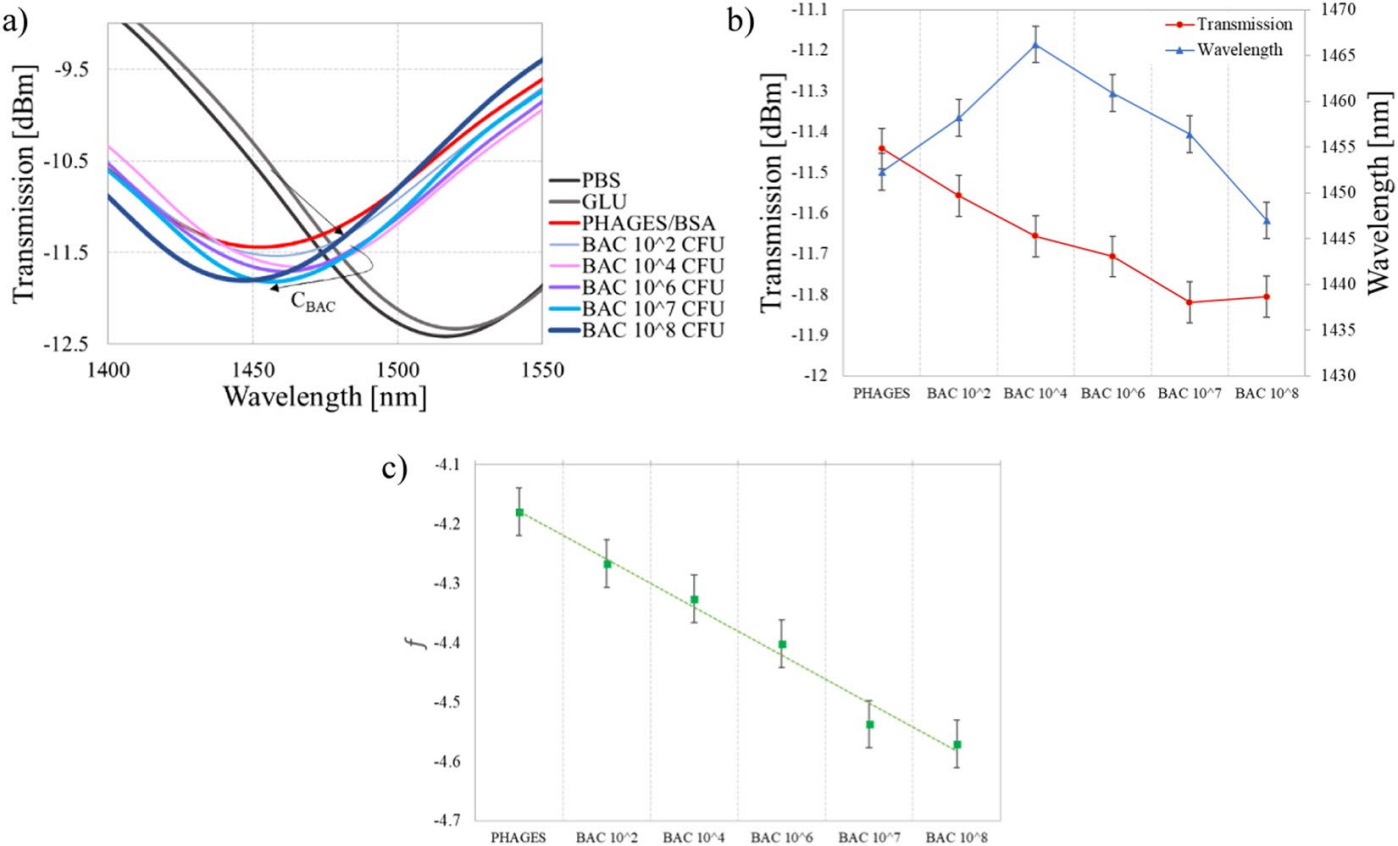
The response of the µIMZI at each stage of the experiment with different concentrations of live *E. coli* C3000 bacteria where (**a**) shows the transmission spectra in PBS after incubation and extensive washing and (**b**) shows a correlation between the transmission and the wavelength (**c**) shows linear combination between the transmission and the wavelength.

The spectra obtained at each stage of the experiment are shown in Fig. 4a. Even for concentration as low as 100 CFU/mL of bacteria, a 5.9 nm spectral shift can be observed. Especially noticeable is the fact that the spectrum shifts differently than during the previous experiment with killed bacteria, what could be expected due to the differences in biolayer properties mentioned above. Subsequent additions of the solutions with a higher concentration of *E. coli* C3000 caused further changes in the wavelength of the transmission minimum. It is worth to mention that the depth of the micromachined cavity is ~62 µm, so it is ~4 µm inside the core. Hence, a layer with the thickness of 4–5 µm will affect *n*_*co*_ the most, creating new conditions for the light propagation inside the core. Further augmentation of the layer exceeding the 4 µm thickness negligibly changes *n*_*co*_, but, in contrast, affects *n*_*cl*_. The addition of bacterial solutions with a concentration of up to 10^4^ CFU/mL shifted the spectrum toward longer wavelengths. It is expected at this stage of the experiment that the *n*_*co*_ is mainly affected, while the RI of bacterial solution stays equal to 1.388 RIU^32^. Considering that the size of bacteria spans form 2 µm^23^ and the length of the fimbriae 1 µm^33^ – the last layer is the thickest one in the system. According to the MS2 infection cycle and place of attachment to the bacterium, we need to take into account that the layer of *E. coli* formed on the sensor surface can increase its density and thickness. Further growth of the adlayer, especially after incubation in a higher concentration of bacteria, affects mainly *n*_*cl*_. Under these conditions according to the Eq. (1), the minimum’s shift changes its direction towards shorter wavelengths. This mechanism explains non-monotonous response. This explanation stays in agreement with the previously presented response of the sensor to increase in Al_2_O_3_ film thickness (Fig. 2b), where the response followed in the same non-monotonic schema.

Despite the non-monotonous response and the initial position of the minimum, the following trials revealed the same trend in a shift of the minimum. For concentrations as small as 10^2^–10^5^ CFU/ml we observed a gradual shift of the minimum towards longer wavelengths and increase of its depth. For higher concentrations −10^6^–10^8^ CFU/ml - the direction of the shift has changed towards shorter wavelengths, but its depth kept increasing. With a combination of these two parameters, i.e., the minimum’s wavelength and transmission, the distinction between different concentrations of bacteria is possible (Fig. 4b). Furthermore, with a linear combination of the transmission and the wavelength which gives a monotonic function, it is possible to obtain an unambiguous mapping of the two values to specific bacteria concentration. An example of such linear combination can be Eq. 2:

2 $$f=A\cdot Wavelength+B\cdot Transmission,$$

where A and B are scaling factors expressed in [nm^−1^] and [dBm^−1^], respectively. The factors transform wavelength and transmission into the same range and guarantee the consistency of units. Function given in Eq. 2 with appropriate coeficients simplify the recognition of the bacteria concentration. As an example we can substitute A = 0.005 and B = 1, the outcome is shown in Fig. 4c.

**Figure 5:**
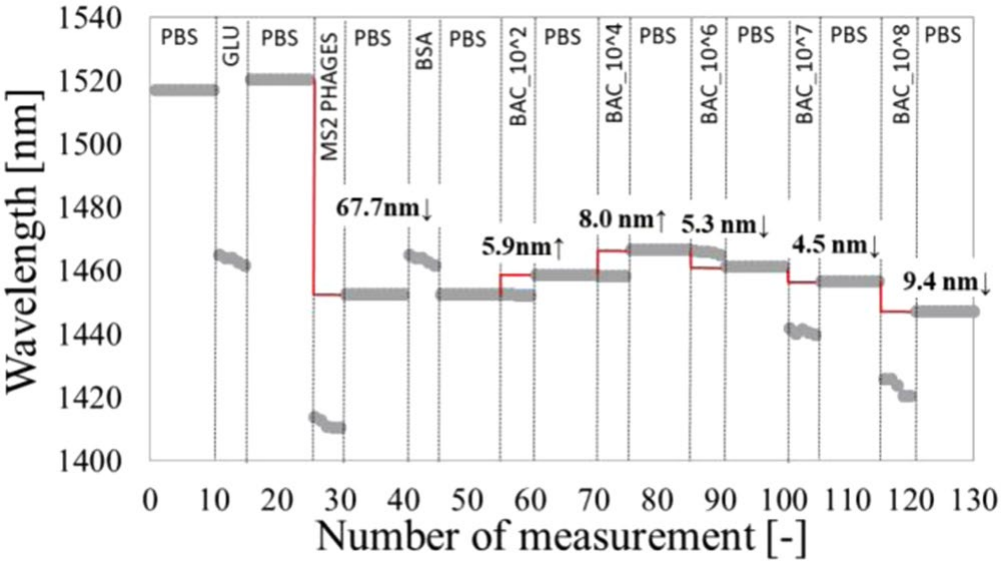
A shift of the minimum’s wavelength at each stage of the experiment induced by functionalization and incubation in different concentrations of live bacteria.

To assure that the obtained effects come from the formation of the biological layer and not from changes in the RI of the solution, the wavelength of the minimum after each stage of the biofunctionalization is plotted (Fig. 5). Due to higher *n*_*cl*_ present in the cavity during the functionalization process, e.g., incubation with GLU, the spectrum temporary experienced shift towards lower wavelength. We can observe the same effect during incubation with, i.e., phages, or higher concentrations of bacteria (10^7^ and 10^8^ CFU/mL). After extensive washing of the surface with PBS, the signal changes, revealing the influence of the adlayer.

The described sensor has shown superiority over the other fiber-optic platforms regarding sensitivity, easiness of fabrication, and the required volume of the sample. The similar assay strategies using APTES/GLU surface functionalization and phages were used for detection of other *E. coli* strains by employing LPFG^7,22^, FBG^34^, and multimode microfiber probe^35^. Although the detection limit of these probes reached 10^3^ CFU/ml, sensitivities of those platforms were lower. The similar strategy using antibodies as a biorecognition element was used to detect other *E. coli* strains by employing plastic optical fiber^36^. It resulted in a LOD of 10^3^ CFU/mL with a detection time of 10 minutes per sample, but a sample volume required for measurements was equal to 5–6 ml. The U-bent fiber optic probe^37^ using immobilized antibodies was also developed for *E. coli* detection resulted in a LOD of 10^3^ CFU/ml, using 500 µl of the sample per measurement whereas our method was capable of detecting 100 CFU/ml in over ten times smaller volumes.

### Negative controls

During the adsorption-based measurement, one might expect the *E. coli* bacteria to be easily wiped off the surface by subsequent PBS buffer washes. In the described experiments, however, they remained firmly attached to the fiber surface due to well oriented, immobilized phages. The shown spectral shift was therefore influenced mostly by the change in the thickness of the specific biolayer on the µIMZI surface. To prove this point, two negative control experiments were performed. In the first one to verify the selectivity of the bioreceptor, we used the *B. Thailandensis* strain, which shows no affinity to MS2. The second control experiment was performed with no MS2 phages but using the host C3000 solely. This second experiment aimed to show that without a specific biorecognition element on the surface it is impossible to detect live bacteria just by exposing the biosample to the functionalized surface.

**Figure 6:**
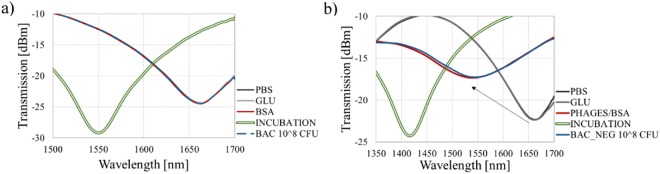
Transmission spectra of the µIMZI at each of the stage of the experiment, showing the response (**a**) for positive *E. coli* C3000 without phages on the sensor’s surface and (**b**) for negative *B. Thailandensis* with MS2 phages.

In both experiments (Figs 5 and 6), it is clear that the minimum during bacteria incubation significantly shifts towards shorter wavelengths (green double curves). This is attributed to the high RI of the highly concentrated bacteria solution (10^8^ CFU/mL). However, after several washes in PBS, despite the very high concentration of the bacteria samples, no wavelength shift was observed, indicating that there is no interaction and no specific binding on the surface. The obtained results confirm our previous findings on the selectivity and reliability of the developed sensor.

## Conclusions

In this work, we show for the first time the capability for monitoring of the growth of a biofilm using a µIMZI-based biosensor. The effect has been shown as a result of exposing the functionalized biosensor to different concentrations of live bacteria. On top of high RI sensitivity (over 15,000 nm/RIU), when using MS2 bacteriophages as recognition elements, the reported device allows for stable and specific label-free detection of alive *E. coli* C3000. The limit of detection of the demonstrated real-time measurements is as low as 100 CFU/mL. The reliability of the functionalized sensor is confirmed with two negative controls. Based on the presented results, it can be concluded that the spectral response is the manifestation of phenomena taking place on the surface of the sensor, and not in the RI of the analyte. During the experiments, the effects of the transitions between surface- and volume-related effects were observed. We believe that the non-monotonic trend in the spectral responses was a result of the investigated working point of the µIMZI as well as the thickness of the biolayer. Due to its very high RI sensitivity, very low-temperature sensitivity and ability to investigate sub-nanoliter volumes, the sensor is well suited for future biological and chemical analysis.
